# How Does the Entrepreneurship Education Influence the Students’ Innovation? Testing on the Multiple Mediation Model

**DOI:** 10.3389/fpsyg.2019.01557

**Published:** 2019-07-10

**Authors:** Xingjian Wei, Xiaolang Liu, Jian Sha

**Affiliations:** ^1^ School of Business Administration, South China University of Technology, Guangzhou, China; ^2^ Youth Development Research Center, Guangxi University, Nanning, China; ^3^ College of Animal Science and Technology, Guangxi University, Nanning, China

**Keywords:** entrepreneurship education, innovation, political skills, entrepreneurial opportunity recognition, multiple mediating effect

## Abstract

This study aims to explore the multiple mediating effects of political skills and entrepreneurial opportunity recognition between perceived entrepreneurship education and innovation. Structural equation is used to analyze data collected from 269 Chinese student entrepreneurs. Results showed that (1) there is a positive relationship between perceptions of entrepreneurship education and perceptions of innovation, (2) political skills and entrepreneurial opportunity recognition individually play a mediating role between perceived entrepreneurship education and innovation, and (3) political skills and entrepreneurial opportunity recognition play a chain mediating effect between perceived entrepreneurship education and innovation.

## Introduction

Entrepreneurship education cultivates innovative talents, which are an important driving force for future development. At present, innovation-driven development strategies place new demands on entrepreneurship education. However, most of the current research and discussion in this field focuses on the construction of teaching staff in the entrepreneurial education ecosystem (Ruskovaara and Pihkala, 2015), curriculum development (Falck et al., 2016), and whether entrepreneurship education can influence the Intention of entrepreneurship (Martin et al., 2013; Pittaway and Cope, 2016). Based on the theory of social cognitive, the individual traits and environmental of learners greatly influence the realization of entrepreneurship education. In-depth study of the mechanism of entrepreneurship education, which drives innovation and development, can further improve the research on entrepreneurship education (Baum et al., 2001; Morris et al., 2013).

Innovation is seen as an internal driver; innovation relates to an entrepreneurial mindset; thus, development of new products or entrance to new markets is the result of entrepreneurship (Miller, 1983; Covin and Slevin, 1989). Entrepreneurship education is an important way for entrepreneurs to acquire resources, enhance innovative ability and innovative personality, and build multi-level learning channels for entrepreneurs by integrating various knowledge and value systems. From knowledge learning to skills improvement, entrepreneurship education includes general ability development and improvement of professional ability. Entrepreneurial competence, which is important for success, mainly refers to the ability to identify opportunities and develop the necessary resources and capital (Arthurs and Busenitz, 2006; Kettunen et al., 2013), in addition to technical, financial, and legal knowledge (Kuratko, 2005). Considering that entrepreneurship ability is diversified, Bacigalupo et al. (2016) build an entrepreneurial competency framework that includes opportunity identification, entrepreneurial skills that represent “resources,” action areas, and 15 competency lists. Gianesini et al. (2018) compared models and classifications of entrepreneurial abilities, arguing that entrepreneurial abilities consist of personality traits, entrepreneurial knowledge, and skills. The research on entrepreneurial ability is increasingly concerned with relevant knowledge and experience to improve skills and develop potential resources to enhance the innovation.

Entrepreneurship education is concerned with fostering creative skills that can be applied in practices, education, and environments supporting innovation (Binks et al., 2006; Gundry et al., 2014). Student entrepreneurs use multi-party interaction to achieve knowledge iteration in the learning network; the innovation process is the result of interactions among the environment, organization, and entrepreneurs (Anderson et al., 2014). Entrepreneurial ability involves adaptive behaviors and strategies to influence others’ actions in relational contexts (Ferris et al., 2005; Tocher et al., 2012), thereby driving innovation and bringing high returns. The entrepreneurship framework by Bacigalupo et al. (2016) considers opportunity identification, entrepreneurial skills, and action as three key areas of entrepreneurial competence. Studies have shown that political skills can help entrepreneurs feel a sense of confidence and control over their work environment. They are likely to be engaged confidently in the dynamics of the environment, and effectively alter attitudes and behaviors to adapt to uncertain conditions (Ferris et al., 2005), with political skills said to explain how individuals recognize opportunities (McAllister et al., 2016). Student entrepreneurs with highly developed political skills can effectively integrate existing resources, accurately identify and interpret social cues from the environment, and gradually become a major force in technology and product innovation. This study selects political skills and entrepreneurial opportunities as mediators to explore how perceived entrepreneurial education influences innovation.

## Theoretical Basis and Hypothesis

Social cognitive theory conceives individuals as agents and active contributors to the development of the circumstances that surround their lives (Bandura, 2018). Individuals are tended to pursue their goals if they consider their own abilities and actions are capable of achieving the desired results (Bandura et al., 2003). Entrepreneurship education helps improve their cognition, constantly adjust their thoughts and actions, and make their entrepreneurship more directional, coherent and meaningful. This study employs the theory of social cognition to examine how learners in entrepreneurship education can enhance their ability to identify opportunities through political skills, which in turn affects entrepreneurs’ innovative awareness, innovative ability, and innovative personality. Learning from observation (Bandura, 1978) to participation (Sims and Sinclair, 2008; Tavella and Franco, 2015), in a network (Berkes, 2009; Chen and Chang, 2014), learning is no longer a single behavior but is implemented in a complex system of relationships. Individuals can transcend immediate circumstances, through self-guidance, shape the present toward the realization of outcomes and goals (Bandura, 2018). General education focuses on the overall development of students, and the entrepreneurial curriculum system lays the foundation for the overall improvement of students’ entrepreneurial ability. From observation to participation, the social learning network provides multi-level learning channels for student entrepreneurs to continuously improve their skills in learning and practice. Therefore, entrepreneurship education might enhance the confidence of the students that he will be able to solve new and unexpected problems.

Skills are described as the ability to apply knowledge in practice, a special ability that drives innovation and development. In entrepreneurship, highly developed political skills can help student entrepreneurs accurately identify and acquire effective resources in a dynamic and complex social environment, as well as create a new combination of technology and knowledge with the support of organizations. Entrepreneur must possess the savviness to effectively understand others in the workplace and adjust their behaviors accordingly. The actual process of opportunity recognition is an interaction between individuals and their environments. Komarkova et al. (2015) argue that skills and opportunities can be used to examine entrepreneurial innovation capabilities. The prior experience and skills of entrepreneurs affect the opportunity recognition process (Dencker et al., 2009; Odia and Odia, 2013). Highly developed political skills transform the resources and experience acquired by entrepreneurship education into the ability to identify and create new products or services; motivate the entrepreneurs to learn together; and enhance innovative awareness, innovative ability, and innovative personality. To deepen the reforms in entrepreneurship education, we have to fully consider the needs and characteristics of student entrepreneurs. Paying attention to the cultivation of students’ entrepreneurial skills is conducive to the realization of the goals of entrepreneurial education organizations, and the overall development needs of entrepreneurial activities.

### The Influence of Perceived Entrepreneurship Education on Innovation

Students’ views on their entrepreneurship education are related to their perception of innovation; fostering innovation through entrepreneurship education is the primary task of universities. Innovative awareness and innovative ability are the core process of students’ innovation activities, which are also influenced by innovation personality. The educational system of universities has to provide an academic environment that may serve as a catalyst for high-technology start-ups (Franke and Lüthje, 2004). If learners are constantly challenged to expand their content knowledge they will be motivated to broaden their cognitive levels (Bandura, 1999), form a defense mechanism to eliminate the negative impact caused by perceived pressure (Granieri et al., 2017). Entrepreneurs are made, not born, by imparting the knowledge and skills needed for a new business venture. The process of shaping the ability of student entrepreneurs is a social interaction process in which information resources are acquired and transformed in the form of observation or direct participation in entrepreneurship education. This process also involves creating new knowledge through transforming experience and putting knowledge into practice. Entrepreneurship education may change a student’s attitudes toward entrepreneurship (Galloway and Brown 2002). Students’ perception and attitudes toward entrepreneurship education can determine whether students’ creativity will be expressed and constitutes a self-judgment of one’s perceived competence in generating novel ideas (Brown and Ulijn, 2004; Beghetto and Kaufman, 2010), forming an internal, lasting, and stable innovative personality. At the same time, entrepreneurship education provides student entrepreneurs with the information, knowledge, and other resources they need, thereby forming a strong atmosphere of innovation and entrepreneurship, reducing environmental uncertainty, and creating a good environment for innovation and development. Entrepreneurship education provides a comprehensive learning management for student entrepreneurs, helping them to establish correct values and cognitive systems, enhance their perceptions of innovation and continuously integrate, and accumulate new knowledge to shape their innovative ability and personality.

*Hypothesis 1*: There will be a positive relationship between perceptions of entrepreneurship education and perceptions of innovation.

### Mediating Role of Political Skills

The primary objective of entrepreneurship education is to develop all essential entrepreneurial skills to meet entrepreneurial success (Lazear, 2004; Audretsch et al., 2016). Traditional entrepreneurial knowledge learning can no longer meet the dynamic environment’s demand for entrepreneurial ability. Entrepreneurship education builds a multi-level social network and comprehensive learning management for the professional ability of entrepreneurs. Entrepreneurship education develops students’ entrepreneurial skills, enabling them to cope with environmental uncertainties and new challenges (Brian and Norma, 2010; Seikkula-Leino, 2011; Premand et al., 2016). Ferris et al. (2000) interpret political skills from four dimensions, namely, networking ability, interpersonal influence, social astuteness, and apparent sincerity. Political skill refers to “*the ability to effectively understand others at work and to use such knowledge to influence others to act in ways that enhance one’s personal and/or organizational objectives*” (Ferris et al., 2005). Politically skilled individuals have superior social astuteness, can help people better understand, and influence others in complex environments, thereby achieving personal and organizational goals (Ferris et al., 2005; Munyon et al., 2015). Political skill helps span boundaries and make up for the shortcomings of social networks on college campuses and facilitate a successful development and usage of network ties (Wei et al., 2012; Fang et al., 2015). Politically skilled individuals are adept at forging relationships with others who have valuable resources and locate themselves in advantageous positions within their social network (Fang et al., 2015). Through social networks, individuals gain access to inaformation, role models, and mentors, establish connections and achieve the esteem and support of peers. The ability of the entrepreneur to gain the trust of others is absolutely essential (Tocher et al., 2015). Since individuals are more willing to openly share knowledge and ideas with those whom they trust (McEvily et al., 2003). Student entrepreneurs with political skills demonstrate problem-solving skills through specific behaviors in relational contexts (Perrewé et al., 2005; Treadway et al., 2013). Highly politically motivated student entrepreneurs can effectively control dynamic and ambiguous environments and make them predictable (Kacmar et al., 2013), and can positively influence innovation by enhancing the personal charm of entrepreneurs (Baron and Tang, 2009). Entrepreneurship education provides multiple channels for student entrepreneurs to obtain resources. The human capital social network built by highly skilled student entrepreneurs enhances the ability of entrepreneurial teams to acquire resources, reduces the cost of resource acquisition, and promotes the willingness of entrepreneurs to share knowledge. With reciprocity, combining access to resources and existing resources, integration generates new knowledge and contributes positively to innovation (Tolstoy, 2009). Therefore, we assumed that political skills would play a mediating role in the associations between perceived entrepreneurship education and innovation.

*Hypothesis 2*: Political skills play a mediating role in the associations between perceived entrepreneurship education and innovation.

### Mediating Role of Entrepreneurial Opportunity Recognition

Entrepreneurs will have to engage in three important tasks, which mainly are opportunity recognition and exploitation, risk taking, and innovating (Chandler and Hanks, 1994). Opportunity recognition is defined as the process of recognizing new and potentially successful ideas (Shane and Eckhardt, 2003), which are influenced by individual characteristics and contextual factors. Entrepreneurship opportunity recognition is the core activity in the early stage of student entrepreneurship; it is the process of correctly understanding and judging market demand, and continuously processing related resources acquired in entrepreneurship learning to shape their innovative ability and personality. Entrepreneurial selecting promises business opportunities, devising, and executing strategies for leveraging them (Chandler and Hanks, 1994). Such competence is often developed experientially through learning by doing (Mitchelmore and Rowley, 2010). Social learning itself is an iterative process of learning, action, reflection, and continuous cooperation. The iterative learning process is considered to be a key component of adapting to environmental changes. In an uncertain entrepreneurial environment, opportunity recognition is becoming a major driver of entrepreneurial behavior (Wang et al., 2013). Student entrepreneurs acquire resources through entrepreneurial education, identify effective knowledge from a large amount of information, integrate processing into new products or services, form new opportunities, improve opportunities for success, and contribute to team creation. We assumed that entrepreneurial opportunity recognition would play a mediating role in the associations between perceived entrepreneurship education and innovation.

*Hypothesis 3*: Entrepreneurial opportunity recognition has a mediating role in the associations between perceived entrepreneurship education and innovation.

### Multiple Mediating Role of Political Skills and Entrepreneurial Opportunity Recognition

Entrepreneurial opportunity recognition, skills, and behaviors together constitute entrepreneurial abilities. Enhancing skills allows entrepreneurs to discover and exploit opportunities that enable they to be more innovative (Fillis and Rentschler, 2010). Political skill is instrumental in gaining access to the information, influence, and referrals necessary for success (Fang et al., 2015). Politically motivated people know what they need to do to succeed, and can make the right actions at the right time to achieve their goals (Blickle et al., 2010). Similarly, we contend that political skill can be used to explain how entrepreneur recognize social influence opportunities (McAllister et al., 2016). High levels of political skill enable entrepreneurs to demonstrate a keen sense of society (Brouer et al., 2011); the social astuteness is conducive to accurately obtaining the key resources needed for entrepreneurship in a dynamic and complex environment. The astute agility of entrepreneurs is a necessary condition for the success of opportunity recognition (Ardichvili et al., 2003). The social network and interpersonal relationships help student entrepreneurs expand the scope of resource acquisition and improve the ability of resource integration. Interpersonal relationships help participants to understand and implement innovative decisions, and improve the efficiency of resource development and product innovation. The apparent sincerity helps entrepreneurs achieve knowledge sharing, and provide a basis for product or service innovation. We assumed that political skills and entrepreneurial opportunity recognition would play a continuous intermediary role in the associations between perceived entrepreneurship education and innovation.

*Hypothesis 4*: Political skills and entrepreneurial opportunity recognition play a continuous intermediary role in the associations between perceived entrepreneurship education and innovation.

**Figure 1:**
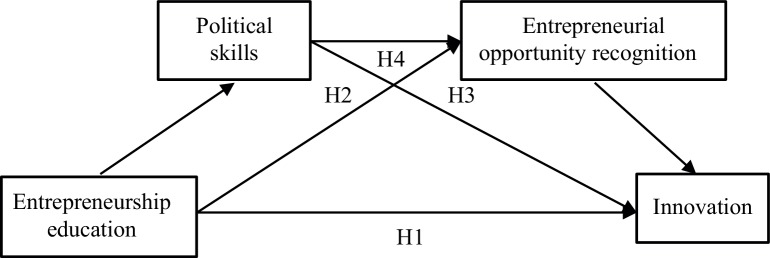
Hypothesized model.

Hypothesized model are shown in Figure 1. Through sharing and cooperation, entrepreneurship education is brought into social learning network from a single level, and completes the acquisition of new knowledge and skills with continuous iterations, enhancing the ability of student entrepreneurs to adapt to changes in the entrepreneurial environment. The entrepreneurial ability of entrepreneurs is considered as a resource in stimulating creativity and the ability to identify opportunities (Kor et al., 2007). Political skill facilitates individuals’ accurate assessment of their work environment and the intentions of others. Driven by innovation, entrepreneurship education constantly improves the path of learning management, and is committed to the improvement of entrepreneurial skills of student entrepreneurs.

## Materials and Methods

### Participants

The participants were recruited from a University located in Nanning, Guangxi Province, China. Questionnaires were distributed to 300 Chinese student entrepreneurs, and 269 valid questionnaires were collected, giving a response rate of 89.67%. Among the respondents, males accounted for 49.17% and females accounted for 50.83%, which shows a relatively balanced gender proportion. Samples from large and medium-sized cities, county-level cities, townships, and rural areas were 30.1, 24.2, 12.3, and 33.5%, respectively. The respondents were all types of students; undergraduate and lower levels accounted for 72.5%, master’s and doctoral students accounted for 27.5%, science and engineering students accounted for 56.9%, followed by economic management accounting for 18.2% and agronomy 11.2%. Among the subjects, 91.1% have been student cadres, and have social networks and interpersonal relationships.

### Procedure

This study selected Chinese student entrepreneurs as the survey object. The participants are involved in entrepreneurial activities such as courses, training programs, and competitions in varying degrees. The participants receive support from teachers and the school with regard to funds, use of venues, and other needs. The survey participants were able to understand the issues involved in this study, thereby meeting the requirements of empirical analysis. In March 2018, we contacted the teachers responsible for entrepreneurship in colleges and universities, to email the participation invitation of this study to their student entrepreneurs. The student entrepreneurs were informed that the data will be used only for research purposes and we will keep their personal information confidential, that participation was voluntary and that they could either refuse to participate in or withdraw from the study at any time. We ask student entrepreneurs to return a form only if they want to participate in the research. The study protocol was approved by the ethics committee of South China University of Technology.

### Measures

To ensure the accuracy of empirical research, this study references important literature published locally and abroad, and selects maturity scales with high reliability and validity. Through in-depth interviews with entrepreneurial team members, this study combines specific scenarios of entrepreneurial education in colleges and universities, and modifies the scales of entrepreneurship education and political skills to make the measurement suitable for student entrepreneurs, finally forming the research scale. This study uses a five-point Likert scale where “1” means “completely inconsistent” and “5” means “very consistent.” The entrepreneurial team members evaluate the corresponding items based on their own real situation.

### Entrepreneurship Education

The entrepreneurship education measured in this study focuses on the perspective of social cognitive from the aspects of environment, organization, and individual learning and behavior. Through interviews with responsible teachers, we can understand the main concerns related to entrepreneurship education in colleges and universities. We start from three aspects: entrepreneurial atmosphere, entrepreneurship curriculum, and entrepreneurial activities. Our main references are Franke and Lüthje (2004) and Qi (2017). To measure the participation of individual entrepreneurs in entrepreneurship education, the education scale has a total of six items such as “A creative university campus atmosphere has inspired your entrepreneurial dream,” “Startup course learning provides the knowledge you need to start a business,” and “The university provides funding for your business, office space, and entrepreneurial tutors”. The Cronbach’s alpha coefficient of this scale was 0.848, indicating that the scale has good reliability.

### Political Skills

The political skills measured in this study are mainly based on the individual level of student entrepreneurs. Based on the work of Ferris et al. (2000), 18 items including networking ability, interpersonal influence, social astuteness, and apparent sincerity were retained for measurement. The results show that the overall Cronbach’s α coefficient of the political skill scale is 0.955, and the scale has good reliability.

### Entrepreneurial Opportunity Recognition

The study draws on Chandler and Hanks (1994) for the measurement of entrepreneurial opportunity recognition and Cai et al. (2014) for the recognition of entrepreneurial opportunities, and measures the ability of student entrepreneurs to identify new opportunities, with four items such as “products and services that can effectively identify customers’ needs”. The results show that the overall Cronbach’s α coefficient of the entrepreneurial opportunity recognition scale is 0.877, indicating that the scale has good reliability.

### Innovation

Miller (1983) was the first to come up with “proactive” innovations and believes innovativeness is one of the important dimensions of entrepreneurial firm. Covin and Slevin (1989) developed a scale to measure the dimensions of innovativeness; the measures involved a mix of traits and attitude. This study agrees with the idea of Naldi et al. (2007) that innovation has a positive relationship with initiative, improved the scale of Covin and Slevin (1989). The higher the subjective initiative of student entrepreneurs, the more obvious is the innovation. The final innovative scale adopts the following items: “I have strong curiosity,” “I like to think and solve problems from multiple angles,” “I always have many new methods and new ideas,” and “I can absorb and apply new ideas faster.” Four items, such as that regarding the “new method,” measure the innovation awareness, innovation ability, and innovation personality of student entrepreneurs. The results show that the Cronbach’s α coefficient of the scale is 0.904, and the scale has good reliability.

### Control Variables

The study controls demographic variables, such as gender and education level of student entrepreneurs, and excludes the possible effects of perceived entrepreneurial education and innovative relationships.

## Results

This study uses SPSS 22.0, AMOS 22.0, and other data analysis instruments. The analysis is divided into three steps: (1) test measurement model including model fit, reliability, and validity test; (2) descriptive statistics on each variable; and (3) we performed multi-mediation tests using the regression bootstrapping method in the PROCESS module (Model 6) developed by Hayes (2013).

### Common Method Deviation Test

This paper uses Harman’s single factor analysis to evaluate the common source variance. Exploratory factor analysis was performed without rotation. The results showed that the variance of the first factor interpretation was 21.694%, and the cumulative interpretation total variance was 50.928%. The first factor explained the variance that was less than half of the cumulative total variance. Therefore, no common method bias effect was observed between the measured variables.

### Confirmatory Factor Analysis

To test the discriminant validity of each variable in this study, a confirmatory factor analysis was performed on each variable using AMOS 22.0 software. The results of Table 1 showed that compared with the single-, two-, and three-factor models, the four-factor model used in this study was the most suitable. The combined effect was ideal, the fitting indexes of the four-factor model were up to standard, and the model fitting degree was good.

**Table 1:** Confirmatory factor analysis results of variable discriminant validity.

Model	*χ*^2^	df	*χ*^2^/df	CFI	GFI	TLI	IFI	NFI	RMSEA
Four-factor model	461.849	336	1.375	0.980	0.907	0.973	0.981	0.932	0.037
Three-factor model	549.356	326	1.685	0.965	0.890	0.950	0.966	0.919	0.051
Two-factor model	585.679	315	1.859	0.957	0.884	0.937	0.958	0.914	0.057
Single-factor model	610.411	308	1.982	0.952	0.876	0.928	0.954	0.910	0.061

*Four-factor model: entrepreneurship education, political skills, entrepreneurial opportunity recognition, and innovation. Three-factor model: entrepreneurship education + political skills, entrepreneurial opportunity recognition, and innovation. Two-factor model: entrepreneurship education + political skills + entrepreneurial opportunity recognition and innovation. Single-factor model: entrepreneurship education + political skills + entrepreneurial opportunity recognition + innovation*.

### Descriptive Statistics and Correlations

The mean, standard deviation, and correlation coefficient of latent variables were statistically analyzed using SPSS 22.0. As shown in Table 2, the mean and standard deviation of each variable were within the acceptable range. According to the correlation coefficient between variables, a significant correlation exists between entrepreneurship education, political skills, entrepreneurial opportunity recognition, and innovation. A significant correlation also exists between gender and entrepreneurship education, and between gender and innovation. The results of descriptive statistics and related analysis preliminarily illustrate the relationship between variables, providing a basis for further data analysis.

**Table 2:** Means, standard deviations, and correlations for variables (*N* = 269).

Variable	Mean	SD	1	2	3	4	5	6
1. Gender	0.509	0.501	1					
2. Education level	0.275	0.447	−0.111	1				
3. Entrepreneurship education	3.910	0.742	−0.168^**^	0.022	1			
4. Political skills	3.811	0.660	−0.117	0.059	0.622^**^	1		
5. Entrepreneurial opportunity recognition	3.841	0.764	−0.103	0.077	0.603^**^	0.777^**^	1	
6. Innovation	4.013	0.714	−0.167^**^	0.073	0.619^**^	0.771^**^	0.781^**^	1

* p < 0.05;

** *p < 0.01*.

### Structural Equation Model Analyses

First, the main effect was tested, with entrepreneurship education as the independent variable and innovation as the dependent variable to construct the structural equation model 1. The fitting index of model 1 meets the requirements (*χ*^2^/df = 2.753, CFI = 0.959, GFI = 0.938, TLI = 0.945, IFI = 0. 959, NFI = 0.937, and RMSEA = 0.081); thus, the model fit is good. The main effect test results show that entrepreneurship education positively affects innovation (*β* = 0.608, *p* < 0.001), and H1 is supported.

**Figure 2:**
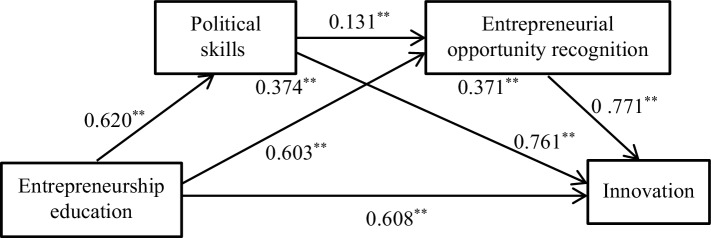
The unstandardized path coefficients in model testing.

Second, models 2 and 3 were established with political skills and entrepreneurial opportunity recognition as single mediators. The results show that the model fits well (Model 2: *χ*^2^/df = 1.002, CFI = 1.000, GFI = 0.905, TLI = 0.998, IFI = 0. 999, NFI = 0.957, and RMSEA = 0.003; Model 3: *χ*^2^/df = 1.490, CFI = 0.989, GFI = 0.958, TLI = 0.982, IFI = 0. 989, NFI = 0.966, and RMSEA = 0.043). Through process V3.1, the bootstrap method was used to repeat the sampling 5,000 times to test the mediating effect. The results are shown in Figure 2. The mediating effect of political skills was 0.374, with 95% confidence interval [0.2983, 0.4534], excluding 0, based on the assumption that H2 was verified. The mediating effect of entrepreneurial opportunity recognition is 0.371, with 95% confidence interval [0.3021, 0.4454], excluding 0, based on the assumption H3 is verified.

Finally, the chain multiple mediation effect was tested. A correlation was observed between the two mediator variables in the political skills and entrepreneurial opportunity recognition. The study assumes that the two variables play a mediating role in the impact of perceived entrepreneurship education on innovation. Therefore, Hayes’ multiple mediation method was used to test the mediating effect. According to process V3.1, the 95% confidence interval of the mediating effect was estimated by extracting 5,000 bootstrap samples, and the chain multi-mediation effect of political skills and entrepreneurial opportunity recognition was tested significantly. The results are shown in Table 3. Entrepreneurship education → political skills → innovative mediating effect is 0.240, 95% confidence interval is [0.1650, 0.3203], excluding 0, and mediating effect is significant. Entrepreneurship education → environmental opportunity recognition → innovation, the mediating effect is 0.082, the 95% confidence interval is [0.0430, 0.1314], excluding 0, and the mediating effect is significant. Entrepreneurship education → political skills → entrepreneurial opportunity recognition → innovative chain multi-mediating effect is 0.131, 95% confidence interval [0.0830, 0.1851], excluding 0, indicating that political skills and entrepreneurial opportunity recognition are between entrepreneurial education and innovation, and H4 is verified.

**Table 3:** Results of the multiple mediation model.

				95% confidence interval	
Effect	Intermediate path	Effect value	Boot standard error	Upper limit	Lower limit
Direct effect	EE → I	0.133	0.045	0.0470	0.2188
Intermediary effect	EE → PS → I	0.240	0.040	0.1650	0.3203
	EE → EOR → I	0.082	0.025	0.0430	0.1314
	EE → PS → EOR → I	0.131	0.026	0.0830	0.1851

*EE, entrepreneurship education; PS, political skills; EOR, entrepreneurial opportunity recognition; I, innovation*.

## Discussion

### Research Conclusions

The study explored the impact mechanism of the influence of perceived entrepreneurship education on innovation based on social cognitive theory. The structural equation model was used to simultaneously test the individual and continuous mediation roles of political skills and entrepreneurial opportunity recognition, and verify the political skills and entrepreneurial opportunity recognition ability of student entrepreneurs. The chain-based multi-mediating role in innovative relationships provides a new path toward considering the impact of perceived entrepreneurial education on the innovation of intermediary mechanisms. The empirical research shows the following results: (1) main effect test. The results show that there will be a positive relationship between perceptions of entrepreneurship education and perceptions of innovation. (2) Intermediary effect test. The test results show that political skills and entrepreneurial opportunity recognition play an intermediary role in perceived entrepreneurship education and innovation, respectively. Political skills enhance the ability to identify entrepreneurial opportunities and play a continuous intermediary role in the impact of perceived entrepreneurship education on innovation.

### Theoretical Implications

The findings inform our understanding of how skills acquired in the entrepreneurship education are associated with innovative awareness, innovative ability, innovative personality, and answer the question of whether entrepreneurship and innovation is perceptible. Entrepreneurship education not only provides human capital such as knowledge and skills but may also transform the attitudes and behaviors of students. For the most part, entrepreneurship education as environmental influences on changing attitudes has been overlooked (Baron, 2006; Medvedeva, 2011). From the social cognitive theory, the research postulates that human behavior is determined by the environmental influences, and description between having capabilities and believing in those capabilities. Individuals are tended to pursue their goals if they consider their own abilities and actions are capable of achieving the desired results. Social cognitive theory conceives individuals as agents and active contributors to the development of the circumstances that surround their lives, through cognitive and motivational, humans can create visualized futures.

We adopt a unique approach in understanding how skills taught within an entrepreneurship education can influence innovation. Base on the social cognitive theory, individuals not only learn skills but also immerse themselves in the entrepreneurial community through entrepreneurship education, which is improving their ability to recognition entrepreneurial opportunities and capture real entrepreneurial opportunities through the community. Entrepreneurial ability is multidimensional and dynamic in nature (Zahra et al., 2006). Skills and entrepreneurial opportunity recognition are the main components of entrepreneurial ability. Explicit political skills based on persuasion, infection, and appeal are the general abilities of entrepreneurs, while entrepreneurial opportunity recognition is the professional skill that entrepreneurs need. Structural equation modeling is used to verify political skills and entrepreneurship opportunity recognition play the multiple mediating role of the relationship between perceived entrepreneurship education and innovation, and clarifies the specific path and internal mechanism of entrepreneurial competence in the impact of perceived entrepreneurship education on innovation. The research results verify that the perceived entrepreneurial education, in the process of shaping the entrepreneurial ability from general to professional, reveals the main factors driving the development of innovation.

### Managerial Implications

As the main body of learning in entrepreneurship education, students should consider their obvious campus characteristics. In student entrepreneurship, many entrepreneurial projects are based on innovative technology transformation and creativity. Innovation is the driving force for the development of entrepreneurial projects. The focus of entrepreneurship education is not on the transfer of theoretical knowledge in the classroom but on the basis of action to improve entrepreneurial professional skills (Kassean et al., 2015). Through participation in learning, student entrepreneurs form a learning network in a good entrepreneurial education environment, use their influence to continuously acquire and exchange valuable resources through persuasion and collaboration, build a shared social resource network, and enhance professional skills. The effectiveness and conversion rate of innovative knowledge strengthens the impact of perceived entrepreneurship education on innovation.

The skills of entrepreneurs can be shaped (Volery et al., 2015) and entrepreneurship education serves as a new incubator of innovative talents, focusing on the improvement of entrepreneurial professional ability. Social cognitive theory can be used to understand the influence of environmental factors on individual innovation awareness, innovative ability and innovative personality. Universities organize and carry out various forms of teaching practice activities; entrepreneurship education enhances the professional competence of students through social learning networks. Student entrepreneurs are regarded as executives with learning and entrepreneurial practices, their high political skills such as good interpersonal relationships, and large social networks can enhance the ability of identify opportunities. Thus, these student entrepreneurs are more likely to become core talents of entrepreneurial teams, playing a role in the impact of perceived entrepreneurship education on innovation.

### Limitations and Future Study Directions

In terms of research samples, owing to the limitations of the research objects, this study only judges the evaluation of entrepreneurship education from the unilateral aspect of the student entrepreneurs and fails to collect the relevant data on the entrepreneurial education managers. Second, considering perception at different times has different influences on human behavior and choice, future studies might consider dynamic tracking from the perspective of organizational managers; research techniques are also biased toward static analysis and are characterized by lack of dynamic tracking. Furthermore, the impact of perceived entrepreneurship education on innovation is multifaceted and multidimensional. In the future, studies can increase the dimensions of research variables in entrepreneurship education and further enrich and develop the research models and conclusions. The present study only considers the mediating factors between entrepreneurial education and innovation. Thus, future research should consider incorporating intermediaries and regulatory factors into the research framework.

## Ethics Statement

This study was carried out in accordance with the recommendations of ethics committee of South China University of Technology with written informed consent from all subjects in accordance with the Declaration of Helsinki. The protocol was approved by the ethics committee of South China University of Technology.

## Author Contributions

XW led the research design, data analysis, and drafted this paper. XL guided the research design and revised the manuscript substantially. JS made contributions in data analysis and paper revision. All authors approved the final version.

### Conflict of Interest Statement

The authors declare that the research was conducted in the absence of any commercial or financial relationships that could be construed as a potential conflict of interest.
